# Sphingolipid Analysis Indicate Lactosylceramide as a Potential Biomarker of Inflammatory Bowel Disease in Children

**DOI:** 10.3390/biom10071083

**Published:** 2020-07-21

**Authors:** Aleksandra Filimoniuk, Agnieszka Blachnio-Zabielska, Monika Imierska, Dariusz Marek Lebensztejn, Urszula Daniluk

**Affiliations:** 1 Department of Pediatrics, Gastroenterology, Hepatology, Nutrition and Allergology, Medical University of Bialystok, 17 Waszyngtona Street, 15-274 Bialystok, Poland; afilimoniuk@op.pl (A.F.); dariusz.lebensztejn@umb.edu.pl (D.M.L.); 2 Department of Hygiene, Epidemiology and Metabolic Disorders, Medical University of Bialystok, 2C Adam Mickiewicz Street, 15-222 Bialystok, Poland; agnieszka.blachnio@umb.edu.pl (A.B.-Z.); m.imierska@gmail.com (M.I.)

**Keywords:** sphingolipid, lactosylceramide, ceramide, Crohn’s disease, ulcerative colitis, inflammatory bowel disease, children

## Abstract

An altered ceramide composition in patients with inflammatory bowel disease (IBD) has been reported recently. The aim of this study was to evaluate the concentrations of sphingolipids in the serum of treatment-naive children with newly diagnosed IBD and to determine the diagnostic value of the tested lipids in pediatric IBD. The concentrations of sphingolipids in serum samples were evaluated using a quantitative method, an ultra-high-performance liquid chromatography-tandem mass spectrometry in children with Crohn’s disease (CD) (n=34), ulcerative colitis (UC) (n = 39), and controls (Ctr) (n = 24). Among the study groups, the most significant differences in concentrations were noted for C16:0-LacCer, especially in children with CD compared to Ctr or even to UC. Additionally, the relevant increase in C20:0-Cer and C18:1-Cer concentrations were detected in both IBD groups compared to Ctr. The enhanced C24:0-Cer level was observed only in UC, while C18:0-Cer only in the CD group. The highest area under the curve (AUC), specificity, and sensitivity were determined for C16:0-LacCer in CD diagnosis. Our results suggest that the serum LacC16-Cer may be a potential biomarker that distinguishes children with IBD from healthy controls and differentiates IBD subtypes. In addition, C20:0-Cer and C18:0-Cer levels also seem to be closely connected with IBD.

## 1. Introduction

**Figure 1:**
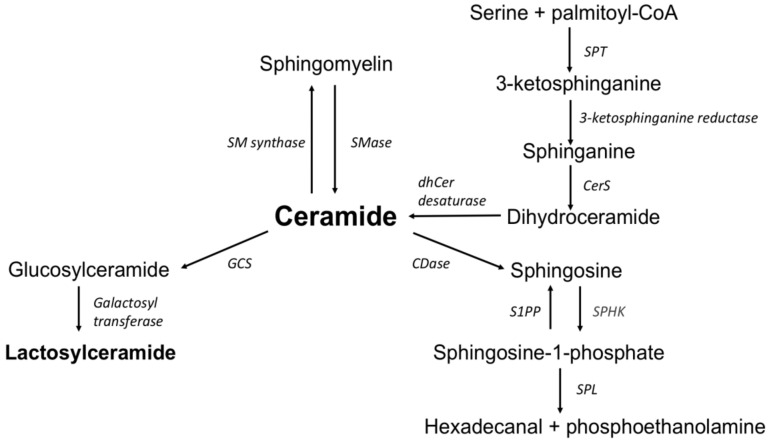
The overview of sphingolipid metabolism. SPT: serine palmitoyltransferase, CerS: ceramide synthase, dhCer desaturase: dihydroceramide desaturase, S1PP: sphingosine-1-phosphate phosphatase, SPHK: sphingosine kinase, SPL: sphingosine-1-phosphate lyase, CDase: ceramidase, SMase: sphingomyelinase, SM synthase: sphingomyelinase synthase, GCS: glucosyl-ceramide synthase.

Inflammatory bowel diseases (IBD) are chronic, relapsing disorders of the gastrointestinal (GI) tract with a not fully understood etiology. There is no single definitive test to confirm the presence of IBD. The diagnostic management takes time and requires performing invasive endoscopic procedures. On the other hand, serum and fecal markers of inflammation are insufficient for discriminating between IBD and other intestinal inflammatory diseases [1,2]. Recently, the applications of some novel techniques such as lipidomics or metabolomics have shown promising results indicating lipid metabolism in IBD pathology and enabling the identification of potential biomarkers [3,4,5]. Ceramide synthesis/degradation seems to be affected in patients with IBD, and may impact the inflammatory status. The overview of sphingolipid metabolism is shown in Figure 1.

Previously, we reported for the first time, that lactosylceramide (C16:0-LacC16) might be a promising marker, allowing us to discriminate children with Crohn’s disease (CD) from patients with ulcerative colitis (UC) and healthy controls in the preliminary study in untargeted metabolomics [3]. Lactosylceramide (LacCer) is thought to be a lipid second messenger in inflammatory disease. LacCer is involved in the activation of cell proliferation, adhesion, migration, and angiogenesis (i.e., in the stages of the inflammatory process, but also cancer development [6,7]. Its expression was detected on the plasma membrane of neutrophils and enhanced intracellular levels were determined in vitro in astrocytes after stimulation with a pro-inflammatory cytokine, tumor necrosis factor-α (TNF-α) [8,9]. Recently, data on altered ceramide composition including LacCer in adult patients with UC have been published, however, there is no report addressing ceramide level in pediatric patients with IBD [5]. The aim of this study was to evaluate the concentrations of sphingolipids including C16:0-LacCer, C14:0-Cer, C16:0-Cer, C18:0-Cer, C18:1-Cer, C20:0-Cer, C22:0-Cer, C24:0-Cer, C24:1-Cer, sphinganine (SPA), and sphingosine (Sph) in the serum of treatment-naive children with newly diagnosed IBD and to determine the diagnostic value of these lipids in IBD by the quantitative method using an ultra-high-performance liquid chromatography-tandem mass spectrometry (UHPLC/MS/MS).

## 2. Materials and Methods 

### 2.1. The Study Groups

The study was conducted on 73 treatment-naive children with newly diagnosed IBD based on ESPGHAN guidelines [1]. The reported symptoms mainly included abdominal pain, diarrhea, the presence of blood in the stool, and the extraintesinal manifestations as weight loss, anemia or erythema nodosum. Among them, CD was diagnosed in 34 cases and UC in 39 cases. The control group (Ctr) included 24 children with normal levels of fecal calprotectin (fCal), a non-invasive marker of IBD, and mainly with functional disorders of the gastrointestinal (GI) tract. None of the patients were being treated at the time of diagnosis and collection of samples for tests. 

The study protocol was approved by the local Ethics Committee (R-I-002/308/2014, R-I-002/294/2018) and conformed to the tenets of the Declaration of Helsinki. Written informed consent was obtained from the parents of all the study participants.

### 2.2. Collection of Samples for Analysis

The biological samples were collected at the time of the IBD diagnosis, shortly after admittance to the department and before administration of any drugs. All patients were fasting on the day of blood collection. Stool sample was collected by the patient/parent 2–4 days prior to the colonoscopy. All collected serum and fecal samples were frozen immediately and stored at a temperature of −80 °C until analysis for serum sphingolipids and fCal. Biological samples were analyzed by an independent researcher who was blinded to all clinical information.

### 2.3. Sphingolipids Measurement 

The concentration of sphingosine (Sph), sfinganine (SPA), ceramides (C14:0-Cer, C16:0-Cer, C18:1-Cer, C18:0-Cer, C20:0-Cer, C22:0-Cer, C24:1-Cer, C24:0-Cer), and C16:0 lactosylceramide (d18:1/16:0) (C16:0-LacCer) in serum was measured according to Bielawski et al. using an ultra-high-performance liquid chromatography-tandem mass spectrometry (UHPLC/MS/MS) approach [10]. Briefly, an internal standard mixture (Sph-d7, SPA-d7, C15:0-d7-Cer, C16:0 -d7-Cer, C18:1-d7-Cer, C18:0-d7-Cer, 17C20:0-Cer, C24:1-d7-Cer, C24-d7-Cer, and C17 Lactosyl Ceramide) (Avanti Polar Lipids, Alabaster, Al) as well as an extraction mixture (isopropanol:ethyl acetate, 15:85; v:v) was added to each sample (100 µL of serum). Samples were then vortexed, sonicated, and centrifuged (5 min. at 3000 g, 4 °C). The supernatants were transferred to the new vials and the pellets were re-extracted. The combined supernatants were evaporated under nitrogen and reconstituted in Solvent B (2 mM ammonium formate, 0.15 % formic acid in methanol). Sphingolipids were analyzed using a Sciex QTRAP 6500 + triple quadrupole mass spectrometer (AB Sciex Germany GmbH, Darmstadt, Germany) equipped with an electrospray ionization source (ESI). Analyses was performed in multiple reaction monitoring (MRM) mode against standard curves, constructed for each compound. The chromatographic separation was performed on a reverse-phase Zorbax SB-C8 column 2.1 × 150 mm, 1.8 μm (Agilent Technologies, Santa Clara, CA, USA) in binary gradient using 1 mM ammonium formate, 0.1% formic acid in water as solvent A, and 2 mM ammonium formate, and 0.1% formic acid in methanol as solvent B at the flow rate of 0.4 mL/min.

### 2.4. Measurement of Blood and Fecal Inflammatory Markers

Serum C-reactive protein (CRP) (mg/L) and albumin (g/dL) levels were determined by immunoturbidimetry (Roche, Hitachinaka, Japan). The complete blood count (CBC) was measured using an automated hematology analyzer (Sysmex, Kobe, Japan). The erythrocyte sedimentation rate (ESR) was evaluated according to the Westergren method (mm/h).

fCal concentration was determined by an enzyme-linked immunosorbent assay ELISA kit (IDK Calprotectin, Immundiagnostik, Bensheim, Germany) in accordance with the manufacturer’s instructions. The assay utilizes the two-site sandwich technique with two selected monoclonal antibodies that bind to human calprotectin. The concentration of calprotectin in each sample was determined directly from the standard curve. The upper normal limit was determined as 65 µg of calprotectin per 1 gram of feces. 

### 2.5. Statistical Analysis

Data were analyzed using Statistica 13 software. The results were presented as median (min–max). The significance of the difference in data was evaluated with the non-parametric Mann–Whitney U-test and the Kruskal–Wallis test by ranks. Spearman’s correlation test was used to analyze the correlations between variables. The diagnostic value of sphingolipid concentration was estimated using receiver operating characteristic (ROC) curve analyses. To calculate the sensitivity, the specificity, the positive predictive value (PPV), the negative predictive value (NPV), and the accuracy of ceramide, the cut-off values were selected based on the Youden index maximization criterion. *p* < 0.05 was considered as statistically significant. 

## 3. Results

### 3.1. Sphingolipid Concentration in Crohn’s Disease, Ulcerative Colitis and Control Group

There were no significant differences regarding age and gender between the study groups. The demographic and clinical characteristics of the study groups are summarized in Table 1. 

**Figure 2:**
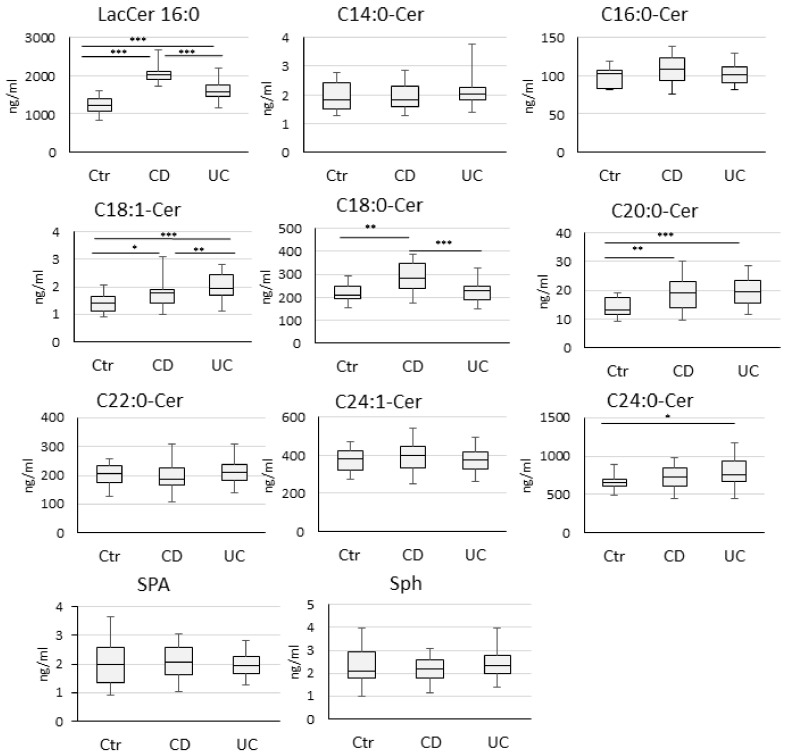
Serum concentrations of sphingolipids in children with Crohn’s disease (CD), ulcerative colitis (UC), and the control group (Ctr). The statistical difference was analyzed by the Mann–Whitney U test. *p* values <0.05 were considered significant. (* *p* < 0.05, ** *p* < 0.01, *** *p* < 0.001).

The serum concentrations of C16:0-LacCer, C18:1-Cer, C18:0-Cer, C20:0-Cer, and C24:0-Cer varied significantly between the IBD and Ctr group (Figure 2, Table S1). 

The largest significant difference in ceramide levels between study groups was determined for C16:0-LacCer. The highest C16:0-LacCer level was noted in children with CD compared to UC and to Ctr. Moreover, the increased concentrations of C20:0-Cer and C18:1-Cer were detected in children with CD or UC compared to Ctr. Additionally, an enhanced C24:0-Cer level was observed only in the UC group, while C18:0-Cer only in the CD group. On the other hand, when comparing results between CD and UC groups, a significant difference in C16:0-LacCer, C18:0-Cer, and C18:1-Cer level was determined (Figure 2, Table S1). 

The area under the ROC curve (AUC) was analyzed to assess the diagnostic value of sphingolipids (C16:0-LacCer, C18:0-Cer, C18:1-Cer, C20:0-Cer, C24:0-Cer), which significantly differentiated patients with IBD from Ctr (CD vs. Ctr, UC vs. Ctr), and CD from UC (Table 2). 

The best results were determined for C16:0-LacCer. A cut-off value of 1724.60 ng/ml discriminated children with CD from the Ctr with 100% sensitivity and 100% specificity (AUC = 1.0). For UC patients, a cut-off level of 1279.72 differentiated these subjects from Ctr with a sensitivity of 97.4% and a specificity of 68.8% (AUC = 0.882, 95%Cl 0.781–982). To distinguish CD from UC patients, the cut-off level of C16:0-LacCer was determined at 1825.4 (AUC = 0.845, 90.6% sensitivity, 74.4% specificity). Evaluation of other ceramides (C18:1-Cer, C18:0-Cer, C20:0-Cer, C24:0-Cer) revealed they were lower, but still diagnostically valuable in distinguishing patients with IBD from Ctr.

There was no difference in C16:0-LacCer, C18:0-Cer, C18:1-Cer, and C20:0-Cer levels related to disease activity in UC as well as CD (Figure S1). Only the C24:0-Cer level was significantly higher in mild compared to moderate/severe UC (Figure S1). With regard to the Paris classification, no differences in ceramide levels were noted in relation to the location and behavior of the disease in both study groups (Table S2).

### 3.2. Correlation between Sphingolipids and the Inflammatory Parameters 

The correlations between sphingolipids that significantly increased in study groups (C16:0-LacCer, C18:0-Cer, C18:1-Cer, C20:0-Cer in CD, and C16:0-LacCer, C18:1-Cer, C20:0-Cer, C24:0-Cer in UC, Figure 2) and CRP, ESR, white blood cell count (WBC), platelet count (PLT), hemoglobin (Hb), fCal, albumin, PCDAI, or PUCAI score, Mayo or SES-CD score are summarized in Table 3. 

In the CD group, all analyzed ceramides and C16:0-LacCer correlated with Hb and albumin. Moreover, C18:0-Cer and C18:1-Cer correlated with PLT. Only C18:0-Cer was additionally associated with WBC and ESR. In UC, C16:0-LacCer was correlated with the majority of inflammatory markers (CRP, WBC, PLT, albumin). None of the laboratory markers correlated with C18:1 or C20:0 in UC patients. Correlations of disease activity scores were noted only with C18:1-Cer in CD and C24:0-Cer in UC. No associations of ceramides with endoscopic scores were found.

## 4. Discussion

Recently, we have reported for the first time, the increased signal detection of lactosylceramide C16:0-LacCer in children with CD in a preliminary study using untargeted metabolomic analysis [3]. In this prospective study, which included a larger group of newly diagnosed treatment naïve children with IBD, we determined the altered serum concentrations of C16:0-LacCer and a few ceramides (C18:0-Cer, C18:1-Cer, C20:0-Cer, C24:0-Cer) using a quantitative method for detection. To our knowledge, there are no available data regarding the measurement of sphingolipid concentration in the serum of patients with CD. In our analysis, the most significant increase in concentration was noted for C16:0-LacCer in children with CD compared to the control group. Furthermore, the levels of C16:0-LacCer in CD were significantly higher than in the UC group, confirming our previous observation in untargeted metabolomics [3]. The sensitivity and specificity of this detection in discriminating IBD patients from the controls reached the highest rate for the CD group and a slightly lower, but still significant rate for UC patients. The only report suggesting the association of lactosylceramide with CD, based on the analysis of the lipid content in intestinal mucosa in scanning densitometry, was published by Stevens et al. The authors revealed an enhanced signal of lactosylceramide in biopsies of patients with CD, while the faint detection of this lipid was found in UC tissue samples, and no presence of this compound was noted in biopsies from uninvolved parts of the bowels of patients with CD, coeliac disease, or microscopic colitis [11]. Since neutrophils, the main source of lactosylceramide, infiltrate both CD and UC inflamed mucosa, the authors suggested that a novel source of LacCer is present in the mucosa or glycosphingolipid metabolism is disturbed in patients with CD [11]. Among the other ceramides analyzed in our study, C18:0-Cer, C18:1-Cer, and C20:0-Cer were significantly increased in the serum of children with CD. Furthermore, a higher level of C18:0-Cer was observed in CD, significantly differentiating this group from UC. To our knowledge, this is the first report of increased concentrations of C18:0-Cer, C18:1-Cer, and C20:0-Cer in CD. Unfortunately, despite the significant correlation of PCDAI with C18:1-Cer, no difference in ceramide levels in relation to disease activity was observed. Only C18:0-Cer correlated with WBC and ESR in the CD group. 

Regarding lipidomics in UC, a few reports concerning adults have been reported recently. In the study by Diab et al., the enhanced signals of very-long-chain ceramides (C24:2-Cer) and (C24:0-Cer) in colon biopsies were determined to be specific for UC (5). In the study by Bazarganipour et al., the content of sphingolipids and ceramides in plasma and colon tissue was estimated using liquid chromatography tandem mass spectrometry (LC-MS/MS) [4]. Interestingly, only C16:0-LacCer and C24:0-LacCer levels were increased in the inflamed tissue when compared to the control tissue. Additionally, the blood level of the most tested ceramides and lactosylceramide types has been shown to be dependent on the disease stage of UC patients. However, in contrast to our study, the concentration of C16:0-LacCer was not increased in plasma when compared to the control group, but the other LacCer type (C18:0 and C24:0) levels were elevated [4]. Additionally, the authors reported enhanced plasma values of C16:0-Cer, C18:0-Cer, and C24:1-Cer, while our analysis did not confirm these findings. Only C20:0-Cer was significantly increased in patients with UC in both studies, though without a differentiation value from children with CD in our report. We also did not note the dependency of ceramide levels on disease activity stage, except the inverse correlation of C24:0 with PUCAI. Interestingly, despite the high C16:0-LacCer concentration in children with UC, and its positive correlation with some blood inflammatory markers, no association with disease activity score was found. The inconsistency of our results compared to the report by Bazarganipour et al. may be related to the differences in the study groups, because our analysis involved treatment-naïve children with UC in opposition to Bazarganipour et al. It is also possible that the ceramide levels change with age and are influenced by medications or coexisting diseases. 

Based on the obtained results, glycerophospholipid metabolism is among the most impacted by the IBD pathway, however many molecular modulations have yet to be explained [3,4,5,12]. In addition to expanding the knowledge about IBD, new discoveries may have practical implications for new targets of treatment and diagnostic purposes.

Interestingly, the marked increase of LacCer and its synthase b-1,4-GalT-V (B4GALT5) expressions were demonstrated in colorectal cancer tissues [7]. Due to the fact that patients with IBD are at an increased risk of CRC, there is a need to find the screening marker that might predict later cancer development in this group of patients. It would be tempting to evaluate in a longitudinal study if abnormal serum LacCer, at the time of IBD diagnosis, could be a screening marker for CRC.

Moreover, LacCer seems to be related to TNF-α, the main pro-inflammatory cytokine involved in the pathogenesis of CD and therapeutic target in this disease. In the study by Pannu et al., TNF-α has been reported to induce LacCer synthesis as well as LacCer synthase activity and astrocyte proliferation [9]. Similar results concerning the increased ICAM-1 expression in endothelial cells, depending on LacCer upregulation following TNF-α stimulation, were demonstrated by Bhunia et al. [13]. The activating effect of LacCer on aortic smooth muscle proliferation has also been observed in atherosclerosis [14]. Since some of the effects of TNF-α are mediated through LacCer synthesis, the latter could serve as a possible target for novel anti-inflammatory therapy in the future or as a marker of anti-TNF-α monoclonal antibody therapeutic effectiveness in IBD.

Although the low number of enrolled patients was the main limitation of our study, the inclusion criterion to include the only newly diagnosed children with IBD was the strength of this study. A well-defined study group containing patients without concomitance to other chronic diseases and therapies allowed for the exclusion of factors potentially affecting the levels of assessed markers of inflammation.

## 5. Conclusions

Our results suggest that serum C16:0-LacCer can be a potential biomarker showing high sensitivity and specificity in distinguishing children with IBD from healthy controls and in differentiating IBD subtypes. In addition, C20:0-Cer and C18:0-Cer serum levels also seem to be closely connected with IBD, especially with CD, however, more research in a larger group of patients is needed. Furthermore, it would be interesting to simultaneously evaluate C16:0-LacCer, C20:0-Cer, and C18:0-Cer concentrations in biological samples (serum, feces, urine) and intestinal tissue to check if systemic levels of these ceramides correspond to the local accumulation of this lipid and depend on therapy. A further study should help to determine whether C16:0-LacCer can be used to monitor the course of IBD.

## Figures and Tables

**Table 1:** Demographic and clinical characteristic of patients with CD, UC, or Ctr. The statistical difference was analyzed by the Mann–Whitney U-test. *p* value < 0.05 was considered significant.

Characteristic	UC	CD	Ctr	*p*
N° of pts	39	34	24	NA
Age (median years)	14 (4–17)	13.5 (6–17)	11.5 (4–17)	NS
Gender (male)	18	22	13	NS
Disease activity index – median (range)	PUCAI 45 (10–75)	PCDAI 25 (5–55)	NA	NA
Paris classification (No. of pts)				
Location - L1/L2/L3	NA	17/3/14	NA	NA
Behavior - B1/B2/B2B3	NA	11/20/3	NA	NA
Growth - G0/G1	NA	22/12	NA	NA
Extent - E1/E2/E3/E4	9/8/9/13	NA	NA	NA
SES-CD	NA	11.5 (0–31)	NA	NA
Mayo endoscopic score I/II/III	6/16/17	NA	NA	NA
ESR (mm/h); median (range)	17 (2–130)	38 (2–120)	2 (2–13)	<0.00001 ^a^
CRP (mg/l); median (range)	3.7 (0.1–299)	24.5 (0.67–342)	0.3 (0.3–1.7)	<0.00001 ^a^
fCal (µg/g); median (range)	1789 (18.6–3405)	1928 (522–3479)	16 (3.8–64)	<0.00001 ^a^

UC: ulcerative colitis, CD: Crohn’s disease, Ctr: control group, PUCAI: pediatric ulcerative colitis activity index, PCDAI: pediatric Crohn’s disease activity index, NA: not applicable, No. of pts: number of patients, L1: distal 1/3 ileum, L2: colonic, L3: ileocolonic, B1: non-stricturing, non-penetrating, B2: stricturing, B2B3: stricturing, penetrating, G0: no evidence of growth delay, G1: growth delay, E1: ulcerative proctitis, E2: left-sided colitis, E3: extensive colitis (hepatic flexure distally), E4: pancolitis, SES-CD: simple endoscopic score for Crohn’s disease, ESR: erythrocyte sedimentation rate, CRP: C-reactive protein, fCal: fecal calprotectin, NS: not significant, ^a^ UC vs. Ctr and CD vs. Ctr.

**Table 2:** Analysis of diagnostic efficiency of selected sphingolipids that significantly differentiated patients with CD from Ctr; with UC from Ctr; and CD from UC. Statistical analysis is described in the “Materials and Methods” section.

Marker	AUC	SE	95% C.I. (AUC)	*p*	Cut-Off	Sensit	Specific	PPV	NPV	ACC
1/ CD vs Ctr										
C16:0-LacCer	1.0	0	(1.0–1.0)	<0.000001	>1724.595	100%	100%	100%	100%	100%
C18:1-Cer	0.694	0.081	(0.535–0.854)	<0.05	>1.375	84.4%	50.0%	77.1%	61.5%	72.9%
C18:0-Cer	0.783	0.067	(0.652–0.915)	<0.00001	>226.648	87.5%	62.5%	82.4%	71.4%	79.2%
C20:0-Cer	0.736	0.071	(0.597–0.876)	<0.001	>19.206	50%	100%	100%	50%	66.7%
2/ UC vs Ctr										
C16:0-LacCer	0.882	0.051	(0.781–0.982)	<0.000001	>1279.717	97.4%	68.8%	88.1%	91.7%	88.9%
C18:1-Cer	0.826	0.059	(0.711–0.941)	<0.000001	>1.66	78.9%	75.0%	88.2%	60.0%	77.8%
C24:0-Cer	0.694	0.073	(0.55–0.838)	<0.01	>710.722	63.2%	81.3%	88.9%	48.1%	68.5%
3/CD vs UC										
C16:0-LacCer	0.84	0.049	(0.745–0.936)	<0.000001	>1825.359	90.6%	73.7%	74.4%	90.3%	81.4%
C18:1-Cer	0.673	0.065	(0.547–0.8)	<0.01	>1.914	75.0%	57.9%	60.0%	73.3%	65.7%
C18:0-Cer	0.787	0.055	(0.679–0.895)	<0.00001	>263.367	65.6%	86.8%	80.8%	75.0%	77.1%

CD: Crohn’s disease, Ctr: control, UC: ulcerative colitis, AUC: area under the curve, SE: standard error, C.I: confidence interval, Sensit.: sensitivity, Specific.: specificity, PPV: positive predictive value, NPV: negative predictive value, ACC: accuracy.

**Table 3:** Correlations of sphingolipids with some inflammatory markers and disease activity scores in CD and UC (Spearman’s rank correlation analysis).

Variables	C16:0-LacCer	C18:0-Cer	C18:1-Cer	C20:0-Cer	C24:0-Cer
**CD group**					
CRP	*R* = 0.198 *p* = 0.25	*R* = 0.136 *p* = 0.46	*R* = 0.038 *p* = 0.83	*R* = 0.109 *p* = 0.55	NA
ESR	*R* = 0.253 *p* = 0.15	*R* = 0.580 *p* = 0.0007	*R* = 0.344 *p* = 0.06	*R* = 0.271 *p* = 0.15	NA
Albumin	*R* = −0.377 *p* = 0.036	*R* = −0.481 *p* = 0.006	*R* = −0.433 *p* = 0.01	*R* = −0.406 *p* = 0.023	NA
WBC	*R* = 0.013 *p* = 0.94	*R* = 0.441 *p* = 0.01	*R* = −0.02 *p* = 0.89	*R* = −0.20 *p* = 0.25	NA
Hb	*R* = −0.451 *p* = 0.01	*R* = −0.432 *p* = 0.01	*R* = −0.410 *p* = 0.02	*R* = −0.507 *p* = 0.003	NA
PLT	*R* = 0.323 *p* = 0.07	*R* = 0.626 *p* = 0.0001	*R*= 0.548 *p* = 0.001	*R* = 0.099 *p* = 0.59	NA
fCal	*R* = 0.070 *p* = 0.69	*R* = 0.293 *p* = 0.10	*R* = 0.147 *p* = 0.42	*R* = 0.127 *p* = 0.49	NA
PCDAI	*R* = 0.252 *p* = 0.14	*R* = 0.261 *p* = 0.15	*R* = 0.382 *p* = 0.03	*R* = 0.009 *p* = 0.959	NA
SES-CD	*R* = 0.109 *p* = 0.56	*R* = 0.057 *p* = 0.75	*R* = 0.09 *p* = 0.61	*R* = −0.05 *p* = 0.755	NA
**UC group**					
CRP	*R* = 0.442 *p* = 0.005	NA	*R* = 0.054 *p* = 0.74	*R* = 0.099 *p* = 0.55	*R* = −0.226 *p* = 0.17
ESR	*R* = 0.200 *p* = 0.23	NA	*R* = 0.226 *p* = 0.19	*R* = 0.122 *p* = 0.48	*R* = −0.127 *p* = 0.46
Albumin	*R* = −0.320 *p* = 0.049	NA	*R* = −0.05 *p* = 0.73	*R* = −0.183 *p* = 0.27	*R* = 0.217 *p* = 0.19
WBC	*R* = 0.358 *p* = 0.027	NA	*R* = 0.054 *p* = 0.74	*R* = 0.135 *p* = 0.41	*R* = −0.352 *p* = 0.03
Hb	*R* = −0.165 *p* = 0.319	NA	*R* = −0.251 *p* = 0.12	*R* = −0.029 *p* = 0.85	*R* = −0.01 *p* = 0.91
PLT	*R* = 0.403 *p* = 0.012	NA	*R* = 0.271 *p* = 0.09	*R* = 0.284 *p* = 0.08	*R* = −0.166 *p* = 0.31
fCal	*R* = 0.121 *p* = 0.46	NA	*R* = 0.077 *p* = 0.64	*R* = −0.043 *p* = 0.79	*R* = −0.296 *p* = 0.07
PUCAI	*R* = 0.053 *p* = 0.74	NA	*R* = −0.09 *p* = 0.58	*R* = 0.147 *p* = 0.37	*R* = −0.341 *p* = 0.03
Mayo score	*R* = −0.13 *p* = 0.43	NA	*R* = −0.149 *p* = 0.37	*R* = −0.05 *p* = 0.72	*R* = −0.126 *p* = 0.44

UC: ulcerative colitis, CD: Crohn’s disease, PUCAI: pediatric ulcerative colitis activity index, PCDAI: pediatric Crohn’s disease activity index, CRP: C-reactive protein, fCal: fecal calprotectin, ESR: erythrocyte sedimentation rate, Hb: hemoglobin, WBC: white blood cell count, PLT: platelet count, NA: not applicable.

## Supplementary Materials

The following are available online at https://www.mdpi.com/2218-273X/10/7/1083/s1, Figure S1: Distribution of sphingolipids for clinical activity in children with ulcerative colitis (UC) (A) and Crohn’s disease (CD) (B). Levels of sphingolipids are presented as a fold change relative to control group (Ctr). The statistical difference was analysed by Mann-Whitney U test. P values <0.05 were considered significant. ** *p* < 0.01. Table S1: Serum concentrations (median ng/mL; min-max) of selected ceramides and C16:0-LacCer which significantly differentiated patients with CD from Ctr; with UC from Ctr; and CD from UC. Statistical analysis is described in the “Materials and Method” section., Table S2: Differences in the serum concentration (median ng/ml; min-max) of selected ceramides (C18:1-Cer, C18:0-Cer, C20:0-Cer for CD and C18:1-Cer, C20:0-Cer, C24:0-Cer for UC) and C16:0-LacCer in relation to Paris classification. The Kruskal-Wallis test by ranks was used in statistical analysis.
